# Desire to delay the first childbirth among young, married women in India: a cross-sectional study based on national survey data

**DOI:** 10.1186/s12889-020-8402-9

**Published:** 2020-03-18

**Authors:** Ismael Ibarra-Nava, Vikas Choudhry, Anette Agardh

**Affiliations:** 1 Social Medicine and Global Health, Department of Clinical Sciences, Lund University, Jan Waldenströms gata 35, 214 28 Malmö, Sweden; 2 Sambodhi Research and Communications Pvt. Ltd., C-126, C Block, Sector 2, Noida, Uttar Pradesh 201301 India

**Keywords:** Young women, India, Intimate partner violence, First childbirth

## Abstract

**Background:**

Young women in India continue to face diverse challenges that threaten their health and wellbeing. The reproductive health and rights of newly married women, who are often expected to begin childbearing soon after marriage, are often neglected. The present study aims to understand some of the factors associated with the desire to delay the first childbirth in young, married women in India.

**Methods:**

The study utilised the data from the most recent National Family Health Survey 2015–16 in India. Our study sample was restricted to married women who were 15–24 years of age and who had never been pregnant at the time of the survey. Chi-squared tests, independent t-tests and multivariable logistic regression analyses were performed to measure associations between multiple independent factors and the reported preferred waiting time for the first childbirth.

**Results:**

Among never pregnant, married women aged 15–24, 21.49% reported a preferred waiting time for their first childbirth of 2 years or more. Belonging to an other backward class, or OBC, (OR_adjusted_ 1.55, 95%CI 1.14–2.10), having completed higher education (OR_adjusted_ 2.04, 95%CI 1.11–3.76), marrying after the age of 18 (OR_adjusted_ 1.57, 95%CI 1.10–2.24), a husband’s higher education level (OR_adjusted_ 2.42, 95%CI 1.27–4.64), a younger husband (OR_adjusted_ 0.75, 95%CI 0.66–0.84) and non-exposure to physical violence (OR_adjusted_ 1.84, 95%CI 1.09–3.11) were significantly associated with a longer preferred waiting time for the first childbirth.

**Conclusion:**

Intimate partner violence and partner characteristics play a role in the childbearing intentions of young women after marriage. Delaying the first childbirth could improve women’s educational and economic opportunities, their health, and the health of their future and properly planned children. To achieve this, it is crucial to promote and respect women’s right to decide who and when to marry, when to have children, and to promote relationships free of gender-based violence.

## Background

As of 2015, young people aged 15–24 years made up around 17% of the world’s population, and this number is expected to increase by 2030 [1, 2]. They comprise a large proportion of the population in many low- to middle-income countries (LMICs). Young people could greatly contribute to the development of LMICs over the next few decades, but this will remain a challenge unless young people gain access to high quality education and better employment opportunities [3]. An integral part of this challenge for young people involves realising their sexual and reproductive health and rights (SRHR). Improving young people’saccess to sexual and reproductive health care services and age appropriate sexuality education can help them avoid early and unintended pregnancies and sexually transmitted infections [3].

SRHR issues unequally affect women due to gender inequities around the globe, which leaves women, particularly young women, in a disadvantaged social position [3]. Their lower social status within their families and communities often undermines young women’s decisions about whether, when, and number of children to have, resulting in unwanted and early pregnancies and restricted educational and economic opportunities [3]. It is estimated that 5 to 33% of women between 15 to 24 years of age have to drop out of school in LMIC countries because of early pregnancy or marriage [4]. Consequently, many young women remain economically dependent on their spouses or their families and confined to their homes to take care of the household and their children.

In India, childbirth most often occurs within marriage. Young married women are expected to begin childbearing and its associated responsibilities soon after co-habitation has begun. In fact, current patterns and trends in early childbearing after marriage continue to encourage rapid population growth in India [5]. This is particularly worrisome in a country of 1.25 billion people, where young people constitute almost one third of the population, and where the proportion of married young people is very high. As per the latest national family health survey (NFHS-4), around 1.6 and 24.4% of men aged 15–19 and 20–24, respectively, are currently married [6]. However, the proportion of currently married women in the same age groups is much higher, with 15.2% of women aged 15–19 and 65.3% of women aged 20–24 being currently married [6]. Furthermore, the proportion of women having unmet need for family planning is highest among women aged 15–19 and 20–24 years, with 22.2, and 22.3%, respectively, compared to the national average of 13% [6]. Marrying young, however, should not mean young couples should have children as soon as possible.

During the past decades, India has focused enormous efforts to prevent early marriage and early childbearing among young women. Despite some improvements in increasing the average age of marriage, early marriage and early childbearing practices are still prevalent and place a burden on young women’s lives. According to the NFHS-4, 27% of women aged 20–24 married before the age of 18 [6]. Marriage before the legal age of 18 is more prevalent amongst poor, less educated women living in rural areas and in the central and eastern regions of India [6]. Its highest prevalence among these groups suggests that early marriage is perpetuating the poverty cycle as women’s educational and economic development is hindered [7]. However, childbearing at a young age can still occur among young, married women who marry after the age of 18.

In research and in practice, the term “early childbearing” is used in relation to young women aged 19 or lower. However, childbearing among young women aged 20–24 could still be seen as a major concern, as demands for higher educational attainment from young people are higher than ever before. Some authors even suggest that these social changes, along with biological ones, should extend our concept of adolescence until the age of 24 [8]. This conceptual change could promote better opportunities for education, empowerment, and future employment, particularly for girls [8]. In India, challenging the appropriate age for women to have children could promote these opportunities.

Most evidence regarding the consequences of early childbearing concerns women aged 15–19. Women in this age group are less prepared for pregnancy than women in their twenties. Adolescent pregnancy has been associated with low birth weight, preterm labour, and poor neonatal outcomes including high rates of early neonatal mortality [9, 10]. While maternal mortality rates are lower in this age group compared to women over the age of 35, a multi-country study found that India contributes the highest number of adolescent maternal deaths in the world after Nigeria [11]. Furthermore, 23% of the overall burden of disease due to pregnancy and childbirth is accounted for by adolescents, despite the fact that only 11% of all births occur in this age group [4]. Although women aged 20–24 do not have an increased risk of poor pregnancy, foetal, or birth outcomes, married women in this age group currently have the same unmet need for family planning as women aged 15–19 [6]. Furthermore, studies have shown that unintended pregnancies can result in poor antenatal care, poor breastfeeding behaviour, poor child nutrition, and even result in poor child health [12].

The determinants of early childbearing mainly focus on maternal and sociodemographic factors. A review conducted in the United States, for example, found that women whose parents had lower education levels and a lower socioeconomic status were more likely to become adolescent mothers [13]. Similarly, Indian women living in certain geographical areas and in rural areas are more likely to marry early and begin childbearing at a young age [14]. In fact, the birth of the first child usually occurs within 2 years after marriage [14]. These findings suggest that the socioeconomic conditions of young women increase the risk of early childbearing.

Other important determinants of women’s reproductive health and choices are the partner they marry and their exposure to intimate partner violence (IPV). Previous studies in other settings have found that a considerable proportion of couples disagree on the number of children they want [15] and that it is usually the husband who wants a larger family size [16]. Furthermore, if the husband disagrees with family planning, the woman is less likely to use contraceptives [17]. Regarding IPV, two studies in India and Kenya found that women exposed to physical violence are less likely to use contraceptives, more likely to experience unwanted pregnancies, and have a higher fertility rate [18, 19].

Despite increasing evidence implicating the partner’s influence on the reproductive autonomy of women, as well as the influence of IPV on women’s reproductive health and choices [19–22], there is little evidence in India on how these factors affect reproductive choices in married women who have never been pregnant. One population-based study explored the determinants of contraceptive use before the first pregnancy among women age 15–34, and found that religion, caste, education, age, age at marriage, media exposure and geographical region played a role in contraceptive use [23]. Another population-based study conducted in six Indian states found that age, education, caste, living in urban areas, and awareness of family planning methods among others were significantly associated with wanting to delay the first pregnancy [24]. However, much more evidence is needed, especially at the national level, concerning the factors, including IPV, associated with delaying the first childbirth. Understanding how these factors affect young women’s reproductive health and choices can help policy makers and institutions account for these factors in their policies and programmes.

The current cross-sectional, population-based national study aims to understand how sociodemographic characteristics, partner characteristics such as education, age and age difference between spouses, and intimate partner violence are associated with the intention to delay the first childbirth among young, married women in India.

## Methods

### Participants

The present cross-sectional, population-based study is based on the National Family Health Survey (NFHS-4) data, conducted between 2015 and 2016. The NFHS-4 was conducted by the Ministry of Health and Family Welfare (MoHFW) of India, which designated the International Institute for Population Sciences (IIPS) Mumbai as the nodal agency to be responsible for the coordination and technical guidance for the survey, which was based on the DHS methodology. The surveys have been carried out since 1992 and provides state- and national-level estimates, while the latest round (NFHS-4) for the first time provided estimates at the district level, of basic demographic characteristics, reproductive health, maternal and child health, family planning, HIV/AIDS and gender-based violence, among others. Both men and women were interviewed, but for the scope of this study only women respondents have been included. Data on husband’s characteristics were only obtained from women respondents when their husbands were also eligible for interviewing. All women aged 15–49 who were usual members of the selected households or who spent the night before the survey in the selected households were eligible for interviewing. A total of 699,686 women aged 15–49 were interviewed to obtain a nationally representative sample.

Our study participants, however, consisted only of currently married, sexually active, never pregnant women aged 15–24. Never pregnant was defined as women who had never given birth, who are currently not pregnant, and who had never had an abortion, miscarriage or stillbirth. Women were considered sexually active if they had ever had sexual intercourse with their current partners. Women currently married but without a gauna^1^ performed were excluded from the sample. Based on these criteria, our study participants consisted of 16,475 young married women aged 15–24. Women who had missing data on partner and IPV characteristics were excluded from our logistic regression model analysis.

### Sampling

To obtain a nationally representative sample of eligible households, a stratified, two-stage, cluster sampling strategy was used. The survey followed a uniform sampling design for each state; nonetheless, oversampling was required in certain areas to ensure national representativeness. Data on IPV and data on men were collected from a subsample of around 15% of households that were selected for the state-level module. If more than one eligible woman lived in a selected household, only one woman was randomly selected for the IPV module of the questionnaire. The module was only administered if complete privacy was obtained, and it was stopped if the respondent requested it at any time or if someone else was trying to listen to the respondent’s answers. In total, 83,397 women were selected for this module and 79,729 completed it (response rate of 95.6%). Pre-determined weights for IPV were assigned on the subsample for nationally representative estimates at the analytical stage. Further details about the sampling strategy, data collection and survey instruments can be found in the final report of the NFHS-4 and on the Demographic Health Survey (DHS) Program website [6, 25].

### Variable characteristics

In the analysis, the outcome measure was preferred waiting time for the first childbirth. Data from the NFHS-4 dataset for this measure was solely accessible in categorized form according to the following categories: less than 12 months, 1 year, 2 years, 3 years, 4 years, 5 years and 6 years, based on the response to the question “How long would you like to wait from now before the birth of (a/another) child?” For our analysis, the outcome was dichotomised as: 1) a preferred waiting time of less than 2 years, and 2) a preferred waiting time of 2 years or more. The reasons for this were two-fold. First, the mean waiting time in India is 2 years [14]; and, second, because when calculating family planning indicators, such as the unmet need for family planning, women with a need for family planning are those who want to delay their next pregnancy for 2 years [26].

The independent factors included sociodemographic, partner and IPV characteristics. The sociodemographic factors used during the analysis were age group (15–19 years, 20–24 years), age at marriage (younger than 18, 18 or older), place of residence (urban, rural), religion (hindu, muslim, others), caste (scheduled tribe/caste [SC/ST], other backwards class [OBC], general), education level (no education, primary, secondary, higher), wealth index (poorest, poorer, middle, richer, richest), and media exposure to family planning (yes or no). The partner characteristics were education level of husband, age of husband, and spousal age difference, which was calculated by subtracting the age of the respondent from the age of her husband. Women were considered exposed to family planning on media if they had heard or seen a family planning message on radio, television, in a newspaper or magazine, or on a wall painting or hoarding in the past few months. IPV characteristics were exposure to physical, emotional, and/or sexual violence and/or controlling behaviour from husband and each was classified as either “yes” for ever exposed or “no” for never exposed.

Information about IPV from currently married women was obtained by asking them if their current husband ever did any of a series of violent behaviours. Exposure to physical IPV was present if their husband had ever pushed, shook, or threw something at them; slapped them; twisted their arm or pulled their hair; punched them with his fist or with something that could hurt them; kicked, dragged, or beat them up; tried to choke or burn them on purpose; or threatened or attacked them with a knife, gun, or any other weapon. Exposure to sexual IPV was present if their husband had ever physically forced them to have sexual intercourse with him even when they did not want to; physically forced them to perform any other sexual acts they did not want to; or forced them with threats or in any other way to perform sexual acts they did not want to. Exposure to emotional IPV was present if their husband had ever said or done something to humiliate them in front of others; threatened to hurt or harm them or someone close to them; or insulted them or made them feel bad about themselves. Exposure to controlling behaviour was present if their husband was jealous or angry if they talked to other men; frequently accused them of being unfaithful; do not permit them to meet their female friends; tried to limit their contact with their families; insisted on knowing where they are at all times; or did not trust them with any money.

### Statistical analysis

The statistical analysis was done using IBM-SPSS Version 21.0. Contingency tables and a chi-square test of independence were used to determine whether there were any associations between the preferred waiting time for the first childbirth and the respondent’s sociodemographic characteristics (age group, place of residence, religion, caste, education level, wealth index, media exposure to family planning, and age at marriage) the partner’s education level, the age of the husband, and exposure to intimate partner violence (physical violence, emotional violence, sexual violence, and/or controlling behaviour). Independent t-tests were used to determine differences between preferred waiting time for the first childbirth and the means of our continuous variables (partner’s age and age difference between spouses). A *p*-value of less than 0.05 was consider statistically significant. The percentages reported in the descriptive tables were weighted to account for the NFHS-4 survey methodology.

A bivariate logistic regression analysis was performed to calculate the unadjusted odds ratios to determinate associations between sociodemographic, partner and IPV characteristics of the respondents and their preferred waiting time for their first childbirth (less than 2 years vs. 2 years or more). Then, a step-wise multivariable logistic regression analysis was performed to calculate the adjusted odds ratios (OR_adjusted_) between the same variables used for the bivariate logistic regression analysis. The variables selected for the multivariable logistic regression analysis were based on a review of the current literature and all measures were adjusted for simultaneously [23, 24]. Before performing the multivariable logistic regression analyses, a matrix of correlations of estimates was generated and we used a threshold of 0.8 to rule out multicollinearity among our independent variables; none of them were excluded from the model. 95% Confidence Interval (95%; CI) have been used to indicate statistical significance.

### Ethical considerations

Informed consent was obtained from all the participants prior to the administration of the NFHS-4 survey. NFHS-4 data are freely available to the public upon request. We submitted our request stating our aims and objectives and permission was granted to download the women’s dataset.

## Results

A total of 16,475 women between 15 and 24 across India were selected for our study. The proportion of women who wanted to delay their first birth for 2 years or more was 21.49% (See Table 1). Data on caste were only available on 15,915 women, data on partner characteristics were only available for 2822 women, and data on IPV were only available for 1651 women due to methodological reasons (see Methods).

**Table 1:** Distribution of characteristics of young married Indian women by preferred waiting time for first childbirth

	Proportion of women by preferred waiting time for first childbirth N (%)		
	< 2 years 12,934 (78.50)	≥2 years 3541 (21.50)	*p*-value^*^
Age group (years)			< 0.01
15–19	4700 (73.4)	1695 (26.6)	
20–24	8234 (81.4)	1846 (18.6)	
Place of residence			< 0.01
Rural	10,036 (78.7)	2739 (21.3)	
Urban	2898 (76.6)	802 (23.4)	
Religion			< 0.01
Hindu	10,354 (77.2)	3029 (22.8)	
Muslim	1796 (83.3)	342 (16.7)	
Other	784 (81.1)	170 (18.9)	
Caste^a^			< 0.01
SC/ST	4554 (79.7)	1102 (20.3)	
OBC	5691 (78.50)	1642 (21.50)	
General	2239 (75.4)	687 (24.6)	
Education level			< 0.01
No education	2251 (86.4)	347 (13.6)	
Primary	1453 (85.2)	274 (14.8)	
Secondary	7499 (77.1)	2221 (22.9)	
Higher	1731 (71.2)	699 (28.8)	
Wealth index			< 0.01
Poorest	2835 (79.2)	677 (20.8)	
Poorer	3028 (78.9)	811 (21.1)	
Middle	2721 (78.5)	756 (21.5)	
Richer	2372 (78.9)	659 (21.1)	
Richest	1978 (74.8)	638 (25.2)	
Media exposure to family planning			< 0.01
No	4753 (79.9)	1149 (20.1)	
Yes	8181 (77.3)	2392 (22.7)	
Age at marriage (years)			0.30
0–17	3687 (77.7)	1022 (22.3)	
18–24	9247 (78.4)	2519 (21.6)	
Husband’s education level^b^			< 0.01
No education	240 (85.8)	38 (14.2)	
Primary	251 (81.6)	44 (18.4)	
Secondary	1322 (78.1)	358 (21.9)	
Higher	389 (71.6)	167 (28.4)	
Age of husband^b^ (years)			< 0.01
Mean	25.01	23.80	
Age difference^b^(years)			0.01
Mean	4.59	4.22	
Physical IPV^c^			< 0.01
No	1105 (75.2)	306 (24.8)	
Yes	211 (88.1)	29 (11.9)	
Sexual IPV^c^			0.02
No	1248 (76.4)	325 (23.6)	
Yes	68 (85.9)	10 (14.1)	
Emotional IPV^c^			0.12
No	1219 (76.8)	313 (23.2)	
Yes	97 (77.0)	22 (23.0)	
Controlling behaviour^c^			0.99
No	627 (76.6)	165 (23.4)	
Yes	689 (77.0)	170 (23.2)	

^a^Data available only for 15,915 women due to missing values

^b^Data available only for 2822 women’s partners

^c^Data on intimate partner violence (IPV) were only collected from 1651 women out of the total sample

^*^The significance level was *p*-value of less than 0.05

Table 1 presents the results from our chi-square and independent t-tests, as well as the distribution of the preferred waiting time for the first birth, by each sociodemographic characteristic, partner characteristic, and exposure to IPV. Between age groups, a greater proportion of women aged 15–19 (26.6%, *p* < 0.01) would prefer waiting 2 years or more for the birth of their first child compared to women aged 20–24 (18.6%, *p* < 0.01). Women living in urban areas (23.4%, *p* < 0.01), Hindu women (22.8%, p < 0.01), those not belonging to either a SC/ST nor to an OBC (24.6%, p < 0.01), women with higher education (28.8%, p < 0.01), wealthier women (23.4%, p < 0.01) and women exposed to family planning on the media (22.7%, p < 0.01) also reported a longerpreferred waiting time for the birth of their first child. Concerning partner characteristics, the higher the education level of the husband, the greater was the proportion of women preferring to delay their first birth 2 years or more. Furthermore, the mean age of the husband (25.01 vs 23.80, *p* < 0.01) as well as the mean age difference (4.59 vs 4.22, p < 0.01) were higher among women who wanted to have their first birth within the next 2 years. Finally, a larger proportion of women not exposed to physical violence reported a longer preferred waiting time for their first birth than women who had been exposed to physical violence.

For our logistic regression analysis, the *p*-value for the Hosmer-Lemeshow statistic was 0.599 (> 0.05), which indicates a good fit for the data. The value of the Nagelkerke R square (pseudo R square) was 0.185, suggesting that our model is an improvement over a model without any predictors. The results (unadjusted and adjusted odds ratios and 95%CIs) from the bivariate and multivariable logistic regression analyses are shown in Table 2. Before adjustment, all sociodemographic characteristics, except place of residence and age at marriage, were significantly associated with a preferred waiting time for the first birth of 2 years or more. The education level of the husband, the age of the husband, and the age difference between spouses were also all significantly associated before adjustment. Only physical IPV out of all types of IPV was associated with preferred waiting time for the first childbirth before adjustment.

**Table 2:** Logistic regression analyses^b^ of characteristics of women wanting to delay first childbirth ≥2 years

	Unadjusted Odd Ratios		Adjusted Odd Ratios	
	OR	95%CI	OR	95%CI
Age group				
20–24 (ref)	1.00		1.00	
15–19	1.60	1.49–1.73	1.04	0.66–1.65
Place of residence				
Urban (ref)	1.00		1.00	
Rural	1.01	0.92–1.10	1.08	0.78–1.51
Religion				
Hindu (ref)	1.00		1.00	
Muslim	0.65	0.57–0.73	0.71	0.45–1.12
Other	0.74	0.62–0.87	1.51	0.92–2.49
Caste				
SC/ST (ref)	1.00		1.00	
OBC	1.08	0.99–1.17	1.55	1.14–2.10
General	1.28	1.16–1.41	1.15	0.77–1.73
Education level				
No education (ref)	1.00		1.00	
Primary	1.22	1.03–1.45	1.08	0.59–2.00
Secondary	1.92	1.70–2.17	1.46	0.91–2.36
Higher	2.62	2.27–3.02	2.04	1.11–3.76
Wealth index				
Poorest (ref)	1.00		1.00	
Poorer	1.12	1.00–1.25	1.09	0.71–1.67
Middle	1.16	1.03–1.30	1.00	0.64–1.56
Richer	1.16	1.03–1.31	1.22	0.74–2.02
Richest	1.35	1.19–1.52	1.46	0.85–2.51
Media exposure to family planning				
No (ref)	1.00		1.00	
Yes	1.20	1.11–1.30	0.90	0.66–1.21
Age at marriage				
0–17 (ref)	1.00		1.00	
18–24	0.98	0.90–1.06	1.57	1.10–2.24
Husband’s education level				
No education (ref)	1.00		1.00	
Primary	1.10	0.69–1.76	1.03	0.52–2.00
Secondary	1.71	1.19–2.45	1.36	0.77–2.39
Higher	2.71	1.84–3.99	2.42	1.27–4.64
Age of husband	0.91	0.88–0.93	0.75	0.66–0.84
Age difference	0.95	0.92–0.98	1.25	1.10–1.40
Physical IPV^a^				
Yes (ref)	1.00		1.00	
No	2.01	1.33–3.03	1.85	1.09–3.11
Sexual IPV^a^				
Yes (ref)	1.00		1.00	
No	1.77	0.90–3.47	1.25	0.58–2.69
Emotional IPV^a^				
Yes (ref)	1.00		1.00	
No	1.13	0.70–1.82	0.59	0.31–1.09
Controlling behaviour^a^				
Yes (ref)	1.00		1.00	
No	1.06	0.83–1.35	0.96	0.74–1.25

^a^Data on intimate partner violence (IPV) were only collected from 1651 women out of the total sample, thus our model only included data from these women

^b^Unadjusted and adjusted odds ratios with their 95%CIs adjusted for sociodemographic characteristics, partner characteristics and exposure to intimate partner violence

After adjustment, belonging to an OBC (OR_adjusted_ 1.55, 95%CI 1.14–2.10), having completed higher education (OR_adjusted_ 2.04, 95%CI 1.11–3.76), and marrying after the age of 18 (OR_adjusted_ 1.57, 95%CI 1.10–2.24) were the only sociodemographic measures that remained statistically significant. All three of them were positively associated with a preferred waiting time for first birth of 2 years or more. Concerning the partner characteristics, the husband having a higher education level also remained significant after adjustment (OR_adjusted_ 2.42, 95%CI 1.27–4.64). After adjustment, the husband’s age was negatively associated with a preferred waiting time for first birth of 2 years or more (OR_adjusted_ 0.75, 95%CI 0.66–0.84), while the age difference between spouses was positively associated (OR_adjusted_ 1.24, 95%CI 1.10–1.40). Finally, women not exposed to physical violence remained, after adjustment, more likely to want to delay their first child (OR_adjusted_ 1.84, 95%CI 1.09–3.11) compared to women exposed to physical violence, who had lower odds of wanting to delay their first childbirth.

## Discussion

This study, to the best of our knowledge, is the first one to explore the various factors, including IPV, associated with intention to delay the first pregnancy among young, married women across India. Our study shows that when and who people marry, as well as physical violence have an effect on the fertility preferences concerning the first childbirth among young women. Certain sociodemographic characteristics also seem to play a role in women’s reproductive preferences and prospects about the future, which is consistent with previous studies.

Our study showed that young women exposed to physical violence would prefer to have their first child in less than 2 years compared with women who live in a marriage free of physical violence. A potential explanation could be the perception that getting pregnant or “giving” their husbands children could put an end to the physical abuse. However, having children has not been associated with decreasing or eliminating IPV and even though physical violence can decrease during pregnancy in some settings, it could also increase in others [27]. Furthermore, it has been found in previous studies that women with children are less likely to leave or end an abusive relationship due to the perceived detrimental effects this could have on their children, as well as the fear of raising children alone [28, 29]. Programmes focusing on gender-based violence could benefit from targeting young women as soon as possible after marriage before women give birth.

Our study also suggests that when and whom women marry play an important role in delaying the first childbirth. Women who marry after their eighteenth birthday are more likely to want to delay their first childbirth. Early marriages are often associated with higher fertility, earlier childbearing practices, shorter birth intervals, and decreased knowledge and access to family planning methods [30]. According to our results, the husband’s age was negatively associated with women wanting to delay the first birth. Just as women’s desire for children increases with age, so does men’s. Furthermore, women’s and men’s fertily preferences might differ, thus affecting women’s reproductive behaviour and choices. One study found that men tend to want larger families than women [16] and their’s husbands preferences seem to affect behaviours, such as contraceptive use [16].

Our results also highlight not only the importance of women’s education with regard to their fertility preferences, but also the education level of their partners. Studies conducted in India have found that the woman’s education level is associated with delaying the first pregnancy, but also with actual contraceptive use to delay it [23, 24]. A multi-country study on partner’s education did not specifically find associations with delaying the first pregnancy; however, associations with increased contraceptive use as well as other healthy reproductive behaviours were found [31].

While our study shows that a considerable proportion of women want to delay their first birth for more than 2 years, it is important to understand that childbearing intentions are different from the intention to use contraceptives and actual contraceptive use. A longitudinal study in India found that, together, both the intention to delay the first birth and intention to use contraceptives predict actual contraceptive use better [24]. Therefore, it would also be important to understand why one might want to delay the first birth but have no intention to use contraceptives, or vice versa.

There are several limitations to our study. First, the cross-sectional sectional design can only indicate associations between our variables and cannot establish causality. However, sociodemographic and partner characteristics are individual characteristics that cannot be influenced by the desire to delay childbirth; therefore, we can safely assume that they precede our married women’s preferred waiting time for the first childbirth. Nevertheless, when it comes to IPV, we can only establish an association. Intention to delay the first or any birth is not static and is dependent on many factors; therefore, other methodological approaches might be better suited to establish how well intention actually predicts delaying birth. Second, self-reporting of exposure to IPV could lead to bias, as it is a sensitive topic. Previous studies have assessed the methodological limitations of intimate partner violence studies. Asserting control over their wives is common for aggressors, and thus a large proportion of abused women might be excluded from surveys such as the DHS, which leads to underestimation of the true problem [23]. While the response rate for the IPV module was 95%, we lack data on the characteristics of those who refused to participate. In addition, since our sample consists of young women, privacy might have been more difficult to obtain, especially among women under 18. Third, women who had abortions were excluded from our study. This could have certain implications when interpreting our results as women who get abortions are usually trying to delay childbirth; however, we decided to exclude them from our analysis because our intention was to understand which factors influence delaying the first childbirth in women who have never been pregnant. Fourth, data on partner characteristics were limited to a subsample of women whose husbands were also interviewed for the men’s questionnaire of the NFHS-4 survey and data on IPV were also limited to a subsample of women. The missing data could potentially limit our model. However, we calculated the minimum sample size required to have reliable results using Cochran’s formula with a confidence level of 95% and a precision error of 5%. Based on this, our required sample size was 1065 and our model included data on 1651 women [32]. Finally, we chose to perform a forward stepwise logistic regression model, which can be a good exploratory approach. However, our results could be inaccurate as this method often yields confidence intervals for effects and predicted values that are falsely narrow [33].

Our study also has several strengths. While no causal relationship can be established due to the cross-sectional design, we can still observe powerful associations at the country level. Unlike other smaller studies, the study design allows for generalisability across young married women in India who have never been pregnant. Furthermore, our findings can be generalised to a certain extent to similar contexts where women marry young and to where women lack agency and power regarding their reproductive health. Finally, our study further contributes to the growing evidence on IPV and its implications for reproductive behaviour. Further studies would benefit from following young married women over time to assess if intention to delay the first birth for a period longer than 2 years actually results in delaying birth and to assess what are the health, social and economic benefits of doing so in India.

As our findings suggest, the reproductive health and rights of young women in India still face numerous challenges. Young Indian women’s empowerment should be at the centre of public health interventions as they would be more likely to take all aspects of their health into their hands. Empowered women are more likely to start and continue to use contraceptives, more likely to deliver safely and more likely to have a skilled birth attendant once they decide to have children [34]. Women who are victims of IPV are at an increased risk of physical injuries, chronic health conditions, unintended and unwanted pregnancies, HIV/STIs, poor mental health, among others [27]. Unfortunately, these outcomes often remain even after the violence has stopped. Therefore, it is especially important to target young women before their health has been seriously affected. Furthermore, involving men to improve SRHR related outcomes has been shown to increase the effectiveness of interventions that address SRHR issues in LMICs [35, 36].

## Conclusions

Intimate partner violence and partner characteristics play a role in the childbearing intentions of young women after marriage. Often, family planning programmes focus on women after their first birth and talk about the importance of spacing and limiting birth. Delaying the first birth is a way of empowering young married women in India, and it could have beneficial effects on young women’s educational and economic opportunities. Better results could be obtained by involving men in family planning programmes and by challenging the gender and social norms that have an effect on women, men and their families and communities.

## Data Availability

The dataset analysed during the current study cannot be made publicly available by us as it belong to the DHS program, but the same dataset is publicly available upon request.

## Abbreviations

DHS: Demographic health survey
IIPS: Institute of population sciences
IPV: Intimate partner violence
MoHFW: Ministry of health and family welfare
NFHS-4: National family health survey 4
SRHR: Sexual and reproductive health and rights

## Footnotes

1 Gauna is a northern ceremony associated with the consummation of marriage, which could even happen years after marriage. Typically, a couple will not live together until the gauna is performed, as the bride usually remains with her parents before it. Thus, sexual activity is highly unlikely between the couple. Gauna is mostly associated with the practice of child marriage.
